# Gastrointestinal symptoms in invasive pneumococcal disease: a cohort study

**DOI:** 10.1186/s12879-020-05211-3

**Published:** 2020-07-06

**Authors:** Hans Kristian Floeystad, Jacob Dag Berild, Bjoern Jardar Brandsaeter, Didrik Frimann Vestrheim, Dag Berild, Are Martin Holm

**Affiliations:** 1 Department of Internal Medicine, Sorlandet Hospital, Kristiansand, Norway; 2 Norwegian Institute of Public Health, Division of infection control and environmental health, Oslo, Norway; 3 Department of Internal Medicine, Lovisenberg Hospital, Oslo, Norway; 4 Department of Infectious Disease, Oslo University Hospital, Oslo, Norway; 5 Institute of Clinical Medicine, Faculty of Medicine, University of Oslo, Oslo, Norway; 6 Department of Respiratory Medicine, Oslo University Hospital, Oslo, Norway

**Keywords:** Invasive pneumococcal disease, Sepsis, Pneumococcal bacteremia, Gastrointestinal symptoms, Mortality

## Abstract

**Background:**

The study aimed to assess whether gastrointestinal (GI) symptoms at admission are associated with increased short-term mortality in patients with invasive pneumococcal disease (IPD).

**Methods:**

We included all patients with IPD at Aker University Hospital in Oslo, Norway, from 1993 to 2008. Clinical data were registered. Survival data were retrieved from official registries. We used Cox regression and Kaplan-Meier curve to compare mortality within 28 days of admission in patients with and without GI symptoms.

**Results:**

Four hundred sixteen patients were included. Of these, 108 patients (26%) presented with GI symptoms, and 47 patients (11%) with GI symptoms only. Patients with GI symptoms were younger (*p* < 0.001) and had less cardiovascular disease (p < 0.001), pulmonary disease (*p* = 0.048), and cancer (*p* = 0.035) and received appropriate antibiotic treatment later. After adjusting for risk factors, we found an increased hazard ratio of 2.28 (95% CI 1.31–3.97) in patients presenting with GI symptoms. In patients with GI symptoms only there was an increased hazard ratio of 2.24 (95% CI 1.20–4.19) in univariate analysis, which increased to 4.20 (95% CI 2.11–8.39) after multivariate adjustment. Fewer patients with GI symptoms only received antibiotics upon admission.

**Conclusions:**

A large proportion of IPD patients present with GI symptoms only or in combination with other symptoms. GI symptoms in IPD are associated with increased short-term mortality.

## Background

*Streptococcus pneumoniae* is a major cause of mortality and morbidity in adults [1–3]. Invasive pneumococcal disease (IPD), where *S. pneumoniae* is found in sterile sites like blood or spinal fluid, is the most severe form of pneumococcal disease with mortality rates of up to 20% [4, 5]. These patients most often present with symptoms from the pulmonary system, like cough, dyspnea and chest pain combined with fever and chills, but some patients present with gastrointestinal (GI) symptoms such as acute abdominal pain [6], nausea and vomiting [7]. There are also case reports of IPD presenting to hospital with GI symptoms where many have been diagnosed as abdominal or pelvic infections [8–11].

Early detection of IPD is essential because of the high mortality during the first 48 h, and some predictors of a fatal outcome have been identified [12, 13]. To our knowledge, there are no large studies comparing mortality in IPD patients presenting with GI symptoms to patients presenting without GI symptoms. We hypothesize that patients presenting with GI symptoms may have a higher mortality, possibly due to a diagnostic delay as a result of atypical presentation.

## Methods

### Study design

Using a paper-based standardized data collection form we retrieved demographic, clinical, laboratory and microbiological data from hospital charts of all adult patients admitted to Aker University Hospital (AUH) between 1 January 1993 and 31 December 2008 with growth of *S. pneumonia* in blood or spinal fluid, as previously described [4]. During the inclusion period, AUH served an unselected catchment population of about 150,000 adults. The first 3 years, the data were collected retrospectively, and the rest were collected prospectively. If a patient had multiple hospitalizations for IPD, we included only the first hospitalization in this study.

### Antibiotic guidelines

The national antibiotic guidelines for community-acquired pneumonia recommended benzylpenicillin 1,2 g × 4, and for septic patients benzylpenicillin 3 g × 4 plus gentamicin 5-7 mg/kg.

### Data collection

Blood cultures were routinely drawn in patients with a suspected infection and a body temperature higher than 38.5 °C, and in patients with a lower body temperature when a severe infection was suspected. A spinal tap was performed if a central nervous infection was suspected. The included patients had IPD, defined as growth of *S. pneumonia* in either blood culture or spinal fluid. Positive cultures were serotyped at the Norwegian Institute of Public Health (NIPH) by the Quellung reaction using serotype-specific antisera (Statens Seruminstitut, Denmark). There were no changes in the indication for obtaining cultures or in the microbiological methods used for the identification of *S. pneumoniae* during the study period. C-reactive protein (CRP) (mg/L) and white blood cell count (WBC) (10^9^/L), was measured at admission.

We defined GI symptoms as abdominal pain, vomiting, diarrhoea, nausea, hematemesis, melena and/or bloating. All adult patients admitted to hospital in the same period with IPD not having any GI symptoms at presentation to hospital constituted the control population. Cardiovascular disease was defined as valvular disease, cardiomegaly, coronary heart disease, arrhythmia and/or heart failure; diabetes as type 1 or type 2 diabetes mellitus; pulmonary disease as chronic obstructive pulmonary disease, tuberculosis, recurrent pneumonia or asthma; cancer as any type of malignant neoplastic disease; smoking as current smoker; alcohol abuse as drinking more than five alcohol units a day; corticosteroid use as the use of any oral or intravenous corticosteroid except inhalation; and immunosuppression as haematological cancer, corticosteroid use, cytotoxic drug treatment and asplenia.

We retrieved date of death for deceased patients within 28 days of admission to hospital from the civil registration system in Norway.

### Statistical methods

We compared continuous variables using the two-sample t-test, and categorical variables using Pearson’s chi-squared test or Fisher’s exact test.

In mortality analysis, we used univariate and multivariate Cox regression. We also graphically assessed mortality using Kaplan-Meier survival curves. Risk factors known to increase the risk of IPD were chosen as variables [14–16]. We also stratified the regression into two time periods; 1993–1999 and 2000–2008. As this stratification reduced the number of outcomes, we had to reduce the number of variables in the regression to avoid overfitting. Therefore, we only included variables that were statistically significant in the multivariate regression for the whole period. The underlying assumptions for the regression analysis were checked and found to be adequately met. Statistical significance value was set to a *p*-value < 0.05. Statistical analyses were performed using Stata (version 15.0 for Windows).

Ethical approval was received from the Regional Ethic Committee in Oslo.

## Results

Four hundred fifty-four first time admissions for IPD were identified during the study period and included in the study. Thirty-eight patients were excluded due to incomplete data on alcohol (*n* = 24) and/or smoking (*n* = 28), leaving 416 patients for the final analysis. The excluded had a higher proportion of men (60.5% versus 44.0%, *p* = 0.050), but were otherwise similar to the included group (Supplement table 1).

One-hundred and eight (26.0%) patients presented with GI symptoms, of these, 47 (11.3%) presented with GI symptoms only. Compared to patients without GI symptoms, the patients with GI symptoms were younger (mean age 59.1 versus 67.1 years, *p* < 0.001), and tended to be hospitalized for a shorter period (12.8 days versus 16.0, *p* = 0.053). They had a lower proportion of cardiovascular disease (21.3% versus 41.9%, p < 0.001), pulmonary disease (19.4% versus 29.2%, *p* = 0.048), and cancer (11.1% versus 20.1%, *p* = 0.035) (Table 1). There were no significant differences in the occurrence of diabetes, registered smoking habits, use of corticosteroids, immunosuppression, and also no differences in registered consumption of alcohol. Similarly, we found no difference in mean CRP, or the proportion of patients with a WBC < 4 or > 12, at admission. When comparing the 47 patients who presented with GI symptoms only to the group without GI symptoms, we found a similar trend, with the patients without GI symptoms on average being older and having more comorbidities.

**Table 1:** Characteristics of 416 adult patients with invasive pneumococcal disease according to gastrointestinal symptoms at admission to Aker University Hospital between 1993 and 2008

	No gastrointestinal symptoms (308 patients)		Gastrointestinal symptoms (108 patients)		p-value^**a**^	Gastrointestinal symptoms only (47 patients)		p-value^**a**^
**Age (years):**								
***Mean (95% CI)***	67.1	(65.2–69.0)	59.1	(55.6–62.7)	**< 0.001**	55.7	(50.2–61.3)	**< 0.001**
***Median (5–95 percentile)***	70.7	(33.0–89.7)	58.8	(25.9–86.7)		56.2	(23.2–88.4)	
**Days hospitalized**^**b**^**:**								
***Mean (95% CI)***	16.0	(13.9–18.2)	12.8	(10.5–15.1)	0.053	13.5	(9.7–17.3)	0.192
***Median (5–95 percentile)***	10.0	(1–45)	9.0	(11–32)		10.0	(1–35)	
**Male (%)**	137	(44.5)	46	(42.6)	0.734	18	(38.3)	0.426
**Inflammatory markers at admission:**								
***Mean CRP (95% CI)***^**c**^	274.6	(256.2–293.0)	290.0	(257.3–322.7)	0.405	247.1	(205.8–288.5)	0.274
***WBC < 4 or > 12 (%)***^**d**^	209	(69.9)	72	(69.9)	1.000	32	(71.1)	0.869
**Comorbidities (%):**								
***Cardiovascular disease***	129	(41.9)	23	(21.3)	**< 0.001**	9	(19.2)	**0.003**
***Pulmonary disease***	90	(29.2)	21	(19.4)	**0.048**	7	(14.9)	**0.040**
***Cancer***	62	(20.1)	12	(11.1)	**0.035**	5	(10.6)	0.121
***Diabetes***	25	(8.1)	10	(9.3)	0.713	4	(8.5)	1.000
**Risk factors (%):**								
***Steroid use***	32	(10.4)	7	(6.5)	0.231	3	(6.4)	0.598
***Smoking***	127	(41.2)	38	(35.2)	0.269	19	(40.4)	0.916
***Alcohol***	35	(11.4)	11	(10.2)	0.737	6	(12.8)	0.779
***Immunosuppressed***^**e**^	48	(15.6)	11	(10.2)	0.166	4	(8.5)	0.269

^a^compared to no gastrointestinal symptoms

^b^info missing for 2 patients in the no gastrointestinal (GI) symptoms group

^c^info missing for 17 patients in the no GI symptoms, 5 in the GI symptoms, and 2 in the only GI symptoms group

^d^info missing for 9 patients in the no GI symptoms, 5 in the GI symptoms, and 2 in the only GI symptoms group

^e^defined as hematological cancer, oral or intravenous steroid treatment, cytotoxic drug treatment and asplenia

**Fig. 1:**
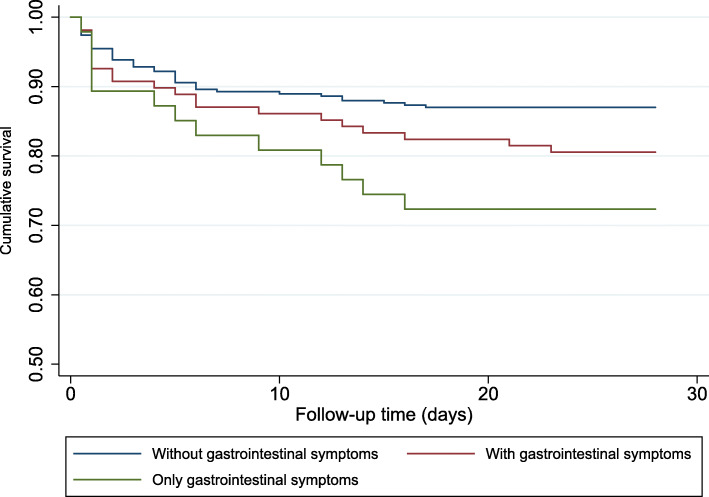
Kaplan-Meier survival curve of adult patients with invasive pneumococcal disease stratified by symptoms at admission to Aker University Hospital, Oslo, Norway, between 1993 and 2008

Sixty-one patients (14.7% of the total study population) died within 28 days of hospital admission. Of the 308 patients without GI symptoms, 40 died (13.0%). Of the 108 patients with GI symptoms, 21 died (19.4%), and 13 of the 47 patients (27.7%) with GI symptoms only died within 28 days. Kaplan-Meier survival curves show an increased 28-day mortality in the group of patients presenting with GI symptoms (Fig. 1). Univariate Cox regression showed that patients presenting with GI symptoms had a non-significantly increased hazard ratio of 1.53 (95% CI 0.90–2.59) (Table 2). However, adjusting for the known risk factors, we found that having GI symptoms upon presentation of IPD significantly predicted death within 28 days of hospital admission, with a hazard ratio of 2.28 (95% CI 1.31–3.97). When stratifying by time periods, this statistically increased mortality was only noted in the early time period with a hazard ratio of 3.93 (95% CI 1.75–8.82) (Supplement table 4). The patients presenting with GI symptoms only had a significantly higher hazard ratio of 2.24 (95% CI 1.20–4.19) in univariate analysis, and 4.20 (95% CI 2.11–8.39) in the multivariate analysis (Table 3). In this group, there was no difference in the hazard ratio between the two time periods (Supplement table 4).

**Table 2:** Univariate and multivariate survival analysis by Cox regression for 416 adult patients with invasive pneumococcal disease at admission to Aker University Hospital between 1993 and 2008

**Univariate analysis**					
***Variables***	***Hazard ratio***	***Std.err***	***p-value***	***95% CI***	
***Gastrointestinal symptoms***	1.53	0.41	0.116	0.90	- 2.59
***Age***	1.03	0.01	**< 0.001**	**1.02**	**- 1.05**
***Being female***	0.60	0.15	**0.046**	**0.36**	**- 0.99**
***Cardiovascular disease***	1.62	0.41	0.061	0.98	- 2.67
***Diabetes***	1.68	0.64	0.169	0.80	- 3.54
***Pulmonary disease***	1.36	0.37	0.255	0.80	- 2.33
***Cancer***	1.73	0.50	0.061	0.98	- 3.06
***Alcohol***	1.53	0.53	0.222	0.77	- 3.01
***Smoking***	1.13	0.29	0.626	0.68	- 1.88
***Steroid***	1.74	0.63	0.126	0.86	- 3.52
***Antibiotics given immediately after blood culture was drawn***	0.49	0.25	0.170	0.18	- 1.36
**Multivariate analysis**					
***Variables***	***Hazard ratio***	***Std.err***	***p-value***	***95% CI***	
***Gastrointestinal symptoms***	2.22	0.63	**0.005**	**1.27**	**- 3.89**
***Age***	1.05	0.01	**0.000**	**1.03**	**- 1.07**
***Being female***	0.52	0.14	**0.016**	**0.31**	**- 0.88**
***Cardiovascular disease***	1.03	0.30	0.905	0.59	- 1.82
***Diabetes***	1.56	0.62	0.259	0.72	- 3.38
***Pulmonary disease***	0.98	0.29	0.955	0.55	- 1.76
***Cancer***	1.58	0.50	0.142	0.86	- 2.93
***Alcohol***	1.89	0.72	0.098	0.89	- 4.00
***Smoking***	1.35	0.38	0.284	0.78	- 2.33
***Steroid***	1.58	0.59	0.226	0.75	- 3.29
***Antibiotics given immediately after blood culture was drawn***	0.62	0.34	0.381	0.22	- 1.79

**Table 3:** Univariate and multivariate survival analysis by Cox regression for 355 adult patients with invasive pneumococcal disease at admission to Aker University Hospital between 1993 and 2008

**Univariate analysis**					
***Variable***	***Hazard ratio***	***Std.err***	***p-value***	***95% CI***	
***Gastrointestinal symptoms only***	2.24	0.72	**0.011**	**1.20**	**- 4.19**
***Age***	1.03	0.01	**0.001**	**1.01**	**- 1.05**
***Being female***	0.66	0.18	0.136	0.39	- 1.14
***Cardiovascular disease***	1.43	0.39	0.192	0.83	- 2.45
***Diabetes***	1.74	0.71	0.173	0.79	- 3.85
***Pulmonary disease***	1.54	0.44	0.135	0.88	- 2.69
***Cancer***	1.45	0.46	0.247	0.77	- 2.71
***Alcohol***	1.32	0.51	0.473	0.62	- 2.79
***Smoking***	1.21	0.33	0.499	0.70	- 2.07
***Steroid***	1.40	0.57	0.407	0.63	- 3.10
***Antibiotics given immediately after blood culture was drawn***	0.46	0.24	0.134	0.17	- 1.27
**Multivariate analysis**					
***Variable***	***Hazard ratio***	***Std.err***	***p-value***	***95% CI***	
***Gastrointestinal symptoms only***	3.96	1.43	**0.000**	**1.95**	**- 8.06**
***Age***	1.05	0.01	**0.000**	**1.03**	**- 1.07**
***Being female***	0.49	0.14	**0.015**	**0.27**	**- 0.87**
***Cardiovascular disease***	1.00	0.31	0.998	0.55	- 1.83
***Diabetes***	1.56	0.67	0.303	0.67	- 3.61
***Pulmonary disease***	1.34	0.43	0.369	0.71	- 2.51
***Cancer***	1.39	0.48	0.342	0.70	- 2.74
***Alcohol***	1.53	0.65	0.315	0.67	- 3.53
***Smoking***	1.34	0.42	0.344	0.73	- 2.46
***Steroid***	1.23	0.52	0.623	0.54	- 2.84
***Antibiotics given immediately after blood culture was drawn***	0.63	0.36	0.419	0.20	- 1.95

In most patients, the focus of infection was assumed to be the lungs. In the group that presented with GI symptoms, a larger proportion was discharged with an unknown focus of infection (Table 4).

**Table 4:** Focus of invasive pneumococcal disease stratified by symptoms for adult patients admitted to Aker University Hospital between 1993 and 2008

Focus at discharge	No gastrointestinal symptoms		Gastrointestinal symptoms		Gastrointestinal symptoms only	
	*n*	*(%)*	*n*	*(%)*	*n*	*(%)*
**Lower respiratory tract**	273	89	87	80	31	66
**Abdominal**	0	0	1	1	0	0
**Central nervous system**	8	3	5	5	4	8
**Other**	7	2	1	1	0	0
**Unknown**	20	6	14	13	12	26
	308		108		47	

All the pneumococcal isolates were penicillin-susceptible. Pneumococcal serotype was identified in 301 (72.4%) of the 416 samples (Supplement table 2). Serotype 1 was the most common in the group presenting with GI symptoms, accounting for 26.5% of the typeable serotypes in patients presenting with gastrointestinal symptoms and 33.3% in patients presenting with GI symptoms only. Serotype 1 was less prevalent in patients not presenting with gastrointestinal symptoms (15.1% vs 26.5 and 33.3%, *p* = 0.023 and *p* = 0.014).

Empirical antimicrobial treatment varied between the groups. There was less use of monotherapy with penicillin in the presence of GI symptoms (32% versus 46%, *p* = 0.013). Also, there was more use of other antibiotics than the combination of penicillin and gentamicin in patients presenting with GI symptoms only compared to no GI symptoms (43% versus 22%, *p* < 0.001) (Supplement table 3). Furthermore, there was a significant difference between the groups in whether or not they received antibiotics immediately after blood culture was drawn. While only 3 % of those without GI symptoms received no antibiotics after blood culture was drawn, 13% of those with GI symptoms only did not (*p* = 0.001). Of the 15 patients that received no antibiotic treatment after blood culture was drawn, ten received penicillin after the blood culture results were available. Among the last five, two were transferred to another hospital within 1 day, and three died within 1, 3 or 7 days, respectively. Including whether antibiotics were given or not immediately after blood culture as a variable in the Cox regression models did not affect the results.

## Discussion

In this study, we found that more than a quarter of patients with IPD presented with GI symptoms at admission. These patients had a higher short-term mortality compared to patients without GI symptoms, despite being younger and having less comorbidities. The increased mortality became even more apparent when restricting the group to those who presented with GI symptoms only. Furthermore, we found that there was a delay in the initiation of adequate antibiotic treatment in the patients presenting with GI symptoms.

### Interpretation

Few studies have investigated GI symptoms in IPD. Watanakunakorn and Bailey found that 10.2% of patients with bacteremic pneumococcal pneumonia had abdominal pain at presentation to the hospital [6]. Their study population is comparable to ours regarding age, comorbidity and mortality, but importantly, only patients with evidence of pneumonia were included in that study, and no characterization of the patients with abdominal pain was given. Weiss et al. found that 33.6% of patients with bacteremic pneumococcal pneumonias had nausea and vomiting necessitating antiemetic treatment or preventing them from eating [7]. Weiss’ study included only bacteremic pneumonias and excluded infectious complications like meningitis, abscesses, empyema, endocarditis and septic arthritis. The study is otherwise comparable to our study, although a further characterization of patients who had nausea and vomiting at admission was not given.

Some studies are supporting our finding that IPD patients with GI symptoms have a higher short-term mortality. In a small study of 38 patients admitted to an intensive care unit with severe pneumococcal bacteremia [17], the in-hospital mortality rate was 89% for the nine patients with GI symptoms and 24% for the 29 patients without gastrointestinal symptoms, i.e. a 3.7 times higher mortality. Although these numbers have to be interpreted with caution due to small sample size, this increased mortality risk is similar to our findings. Another study of 289 patients with unspecified community-acquired infections, found that patients with diarrhoea or vomiting at admission had an increased risk of developing severe sepsis within 48 h of admission [18]. This study might suggest that GI symptoms are not only a marker of poor outcome in IPD but a marker of poor outcome of sepsis in general. However, this study had only 26 positive blood cultures and included all possible foci of infection and microbial agents. Therefore, this study is not directly comparable to ours.

Another explanation for the higher mortality in patients with GI symptoms might be that it could blur the diagnostic evaluation and lead to delayed initiation of adequate therapy. A large proportion of the group with GI symptoms had a focus of unknown origin, which may prolong the time to diagnosis and treatment. More patients with GI symptoms only received no antibiotics early after blood cultures were taken, and if they did, more patients received other antibiotics than penicillin and gentamicin. We do not have data on the exact time to start of antimicrobial therapy in this study, but we know that a reduced time to treatment improves outcome [19]. It is also possible that these patients had an undiagnosed abdominal focus, which could have delayed the initiation of appropriate antibiotic therapy. Several case reports and case series have previously reported abdominal or gynaecological focuses in pneumococcal infections [8–10].

The decision to perform a spinal tap was based on clinical suspicion of meningitis. If there were meningitis cases present misdiagnosed as pneumonia or unknown focus, this might have contributed to an increased mortality in the GI symptoms group as nausea is a common symptom in bacterial meningitis [20]. However, as this was a population with no pneumococcal isolates resistant to penicillin, patients with undiagnosed meningitis would still have been adequately treated with penicillin [21].

In our study, serotype 1 was more prevalent in patients with GI symptoms, and even more prevalent in patients with GI symptoms only. Other studies have found that patients infected by serotype 1 tend to be younger and without significant comorbidity [22–24]. This is in agreement with our finding of less comorbidity in patients with GI symptoms. However, it does not explain the higher mortality, as serotype 1 previously has been associated with a lower mortality rate compared to other serotypes [25].

According to our regression analysis, females had a lower short-term mortality compared to men. When stratifying our analysis into the two time periods, we found that this difference was only significant during the first 7 years. Almost 28% of men died within 28 days in the period 1993–1999, compared to only 11% of women in the same period (supplement table 5). We do not have a good explanation for this increased mortality in men during the first 7 years. There is conflicting evidence in previous studies. For instance, while a higher mortality was found in men than in women with pneumococcal bacteremia [26], a study of severe sepsis and septic shock showed higher mortality in women compared to men [27].

### Limitations

Although it is a strength of the present study that it is a large study of unselected IPD patients with a 16-year long observation time, we did not have complete data on clinical characteristics. Respiratory rate (RR) could have been used to test the association between GI symptoms and severity of sepsis as assessed by using CRB-65, SIRS, SOFA and q-SOFA, and GI symptoms, but RR was only reported in about 40% of the patients. However, we found no difference in inflammatory markers at admission. A second limitation is that data on concurrent severe GI disease and complications were not registered. Gastrointestinal bleeding and perforation are examples of conditions that possibly could have contributed to the increased mortality in the GI symptoms group. Another limitation is the retrospective registration of data using medical records to assess subjective symptoms. However, as the data was collected using predefined forms, we do not believe that this introduced any non-differential measurement bias.

## Conclusions

A large proportion of IPD presents with GI symptoms combined with other symptoms or alone. GI symptoms in IPD is associated with a significantly higher short-term mortality and delayed treatment. It is important to be aware of this atypical presentation of IPD in order to initiate proper treatment rapidly.

## Supplementary information

**Additional file 1 : Table S1.** Characteristics of included and excluded adult patients with invasive pneumococcal disease at admission to Aker University Hospital between 1993 and 2008. **Table S2.** Serotype of invasive pneumococcal disease stratified by symptoms for 416 adult patients admitted to Aker University Hospital between 1993 and 2008. **Table S3.** Empirical antibiotic treatment initiated after blood culture in 416 adult patients with invasive pneumococcal disease admitted to Aker University Hospital between 1993 and 2008. n:number. **Table S4.** Multivariate survival analysis by Cox regression for 416 adult patients with invasive pneumococcal disease at admission to Aker University Hospital between 1993 and 2008 by time period. GI: Gastrointestinal. **Table S5.** Proportion dead within 28 days for 416 adult patients with invasive pneumococcal disease at admission to Aker University Hospital between 1993 and 2008 by sex and time period.

## Data Availability

The data that support the findings of this study are available from the authors upon reasonable request.

## Abbreviations

IPD: Invasive pneumococcal disease
GI: Gastrointestinal
AUH: Aker University Hospital
NIPH: Norwegian Institute of Public Health
CI: Confidence interval
RR: Respiratory rate
