# Supplementary material for: Gastrointestinal symptoms in invasive pneumococcal disease: a cohort study
Source: BMC Infect Dis. 2020 Jul 6;20:479. doi: 10.1186/s12879-020-05211-3 (PMC7339559; doi:10.1186/s12879-020-05211-3)
Supplement: Supplementary file 1 — Additional file 1 : Table S1. Characteristics of included and excluded adult patients with invasive pneumococcal disease at admission to Aker University Hospital between 1993 and 2008. Table S2. Serotype of invasive pneumococcal disease stratified by symptoms for 416 adult patients admitted to Aker University Hospital between 1993 and 2008. Table S3. Empirical antibiotic treatment initiated after blood culture in 416 adult patients with invasive pneumococcal disease admitted to Aker University Hospital between 1993 and 2008. n:number. Table S4. Multivariate survival analysis by Cox regression for 416 adult patients with invasive pneumococcal disease at admission to Aker University Hospital between 1993 and 2008 by time period. GI: Gastrointestinal. Table S5. Proportion dead within 28 days for 416 adult patients with invasive pneumococcal disease at admission to Aker University Hospital between 1993 and 2008 by sex and time period. [file 12879_2020_5211_MOESM1_ESM.docx]

**Supplement table 1**

*Characteristics of included and excluded adult patients with invasive pneumococcal disease at admission to Aker University Hospital between 1993-2008.*

|  | **Included (416 patients)** | | **Excluded due to missing data (38 patients)** | | **p-value** |
| --- | --- | --- | --- | --- | --- |
| **Average age in years (95% CI)** | 65.0 | (63.3-66.7) | 68.3 | (61.4-75.3) | 0.860 |
| **Average days hospitalized (95% CI)*** | 15.2 | (13.5-16.9) | 11.3 | (7.9-14.7) | 0.092 |
| **Male (%)** | 183 | (44.0) | 23 | (60.5) | 0.050 |
| **Inflammatory markers at admission:** |  |  |  |  |  |
| ***Mean CPR (95% CI)^+^*** | 278.6 | (262.6-294.6) | 293.0 | (246.5-339.5) | 0.597 |
| ***White blood cell count <4 or >12 (%)^#^*** | 281 | (69.9) | 31 | (81.6) | 0.130 |
| **Comorbidities (%):** |  |  |  |  |  |
| ***Cardiovascular disease*** | 152 | (36.5) | 19 | (50.0) | 0.101 |
| ***Pulmonary disease*** | 111 | (26.7) | 6 | (15.8) | 0.142 |
| ***Cancer*** | 74 | (17.8) | 9 | (23.7) | 0.368 |
| ***Diabetes*** | 35 | (8.4) | 3 | (7.9) | 1.000 |
| **Risk factors (%):** |  |  |  |  |  |
| ***Steroid use*** | 39 | (9.4) | 1 | (2.6) | 0.233 |
| ***Smoking*** | 165 | (39.7) | 5 | (50.0) | 0.510 |
| ***Alcohol*** | 46 | (11.1) | 3 | (7.9) | 0.206 |
| ***Immunosuppressed^¤^*** | 59 | (14.2) | 3 | (16.2) | 0.456 |
| **info missing for 2 patients in the included group*  *^+^info missing for 14 in the included group*  *^#^info missing for 22 in the included group*  *^¤^defined as hematological cancer, oral or intravenous steroid treatment, cytotoxic drug treatment and asplenia)* | | | | | |

**Supplement table 2**

*Serotype of invasive pneumococcal disease stratified by symptoms for 416 adult patients admitted to Aker University Hospital between 1993-2008.*

| **Serotype** | **No gastrointestinal symptoms** | **Proportion of typable** | **Gastrointestinal symptoms** | **Proportion of typable** | **Only gastrointestinal symptoms** | **Proportion of typable** |
| --- | --- | --- | --- | --- | --- | --- |
| 1 | 33 | 15.1 % | 22 | 26.5 % | 10 | 33.3 % |
| 3 | 8 | 3.7 % | 3 | 3.6 % | 2 | 6.7 % |
| 4 | 32 | 14.7 % | 10 | 12.1 % | 0 | 0.0 % |
| 5 | 0 | 0.0 % | 0 | 0.0 % | 0 | 0.0 % |
| 6A | 7 | 3.2 % | 1 | 1.2 % | 1 | 3.3 % |
| 6B | 2 | 0.9 % | 3 | 3.6 % | 1 | 3.3 % |
| 7F | 16 | 7.3 % | 6 | 7.2 % | 3 | 10.0 % |
| 8 | 8 | 3.7 % | 4 | 4.8 % | 2 | 6.7 % |
| 9N | 6 | 2.8 % | 0 | 0.0 % | 0 | 0.0 % |
| 9V | 18 | 8.3 % | 3 | 3.6 % | 1 | 3.3 % |
| 10A | 6 | 2.8 % | 1 | 1.2 % | 0 | 0.0 % |
| 11A | 2 | 0.9 % | 0 | 0.0 % | 0 | 0.0 % |
| 11B | 0 | 0.0 % | 0 | 0.0 % | 0 | 0.0 % |
| 12F | 6 | 2.8 % | 3 | 3.6 % | 1 | 3.3 % |
| 14 | 24 | 11.0 % | 11 | 13.3 % | 2 | 6.7 % |
| 15B | 1 | 0.5 % | 0 | 0.0 % | 0 | 0.0 % |
| 15C | 0 | 0.0 % | 1 | 1.2 % | 1 | 3.3 % |
| 16F | 4 | 1.8 % | 1 | 1.2 % | 1 | 3.3 % |
| 17F | 2 | 0.9 % | 0 | 0.0 % | 0 | 0.0 % |
| 18C | 3 | 1.4 % | 1 | 1.2 % | 0 | 0.0 % |
| 19A | 4 | 1.8 % | 1 | 1.2 % | 0 | 0.0 % |
| 19F | 4 | 1.8 % | 2 | 2.4 % | 0 | 0.0 % |
| 20 | 1 | 0.5 % | 0 | 0.0 % | 0 | 0.0 % |
| 22F | 6 | 2.8 % | 3 | 3.6 % | 3 | 10.0 % |
| 23A | 1 | 0.5 % | 0 | 0.0 % | 0 | 0.0 % |
| 23F | 13 | 6.0 % | 2 | 2.4 % | 0 | 0.0 % |
| 24F | 2 | 0.9 % | 0 | 0.0 % | 0 | 0.0 % |
| 25A | 1 | 0.5 % | 0 | 0.0 % | 0 | 0.0 % |
| 31 | 1 | 0.5 % | 3 | 3.6 % | 2 | 6.7 % |
| 33 | 1 | 0.5 % | 0 | 0.0 % | 0 | 0.0 % |
| 33F | 2 | 0.9 % | 2 | 2.4 % | 0 | 0.0 % |
| 35F | 2 | 0.9 % | 0 | 0.0 % | 0 | 0.0 % |
| 40 | 2 | 0.9 % | 0 | 0.0 % | 0 | 0.0 % |
| **Typable** | 218 | 70.8 % | 83 | 76.9 % | 30 | 63.8 % |
| **Non-typable/missing info** | 90 | 29.2 % | 25 | 23.2 % | 17 | 36.2 % |
| *Sum* | 308 |  | 108 |  | 47 |  |

**Supplement table 3**

*Empirical antibiotic treatment initiated after blood culture in 416 adult patients with invasive pneumococcal disease admitted to Aker University Hospital between 1993-2008. n:number.*

| **Class of antibiotic:** | **No gastrointestinal symptoms** | | **Gastrointestinal symptoms** | | **p-value*** | **Only gastrointestinal symptoms** | | **p-value*** |
| --- | --- | --- | --- | --- | --- | --- | --- | --- |
|  | *n* | *(%)* | *n* | *(%)* |  | *n* | *(%)* |  |
| **No antibiotic given** | 8 | 3 | 7 | 6 | 0.062 | 6 | 13 | **0.001** |
| **Penicillin ampicillin or dicloxacillin** | 142 | 46 | 35 | 32 | **0.013** | 12 | 26 | **0.008** |
| **Penicillin ampicillin or dicloxacillin combined with gentamicin** | 90 | 29 | 37 | 34 | 0.328 | 9 | 19 | 0.152 |
| **Other** | 68 | 22 | 29 | 27 | 0.220 | 20 | 43 | **<0.001** |
|  | 308 |  | 108 |  |  | 47 |  |  |
| **compared to no gastrointestinal symptoms* |  |  |  |  |  |  |  |  |

**Supplement table 4**

*Multivariate survival analysis by Cox regression for 416 adult patients with invasive pneumococcal disease at admission to Aker University Hospital between 1993-2008 by time period. GI: Gastrointestinal*

| ***Gastrointestinal symptoms (416 patients in total)*** | | | | ***Gastrointestinal symptoms only (355 patients in total)*** | | | |
| --- | --- | --- | --- | --- | --- | --- | --- |
| ***1993-2008 (61 deaths)*** | | | | ***1993-2008 (53 deaths)*** | | | |
| ***Variables*** | ***Hazard ratio*** | ***p-value*** | ***95 % CI*** | ***Variables*** | ***Hazard ratio*** | ***p-value*** | ***95 % CI*** |
| ***GI symptoms*** | 2.11 | 0.007 | 1.23 - 3.63 | ***GI symptoms only*** | 4.00 | 0.000 | 2.04 - 7.84 |
| ***Age*** | 1.04 | 0.000 | 1.02 - 1.06 | ***Age*** | 1.05 | 0.000 | 1.03 - 1.07 |
| ***Being female*** | 0.48 | 0.005 | 0.29 - 0.80 | ***Being female*** | 0.46 | 0.007 | 0.26 - 0.81 |
|  | | | |  | | | |
| ***1993-2000 (30 deaths)*** | | | | ***1993-2000 (25 deaths)*** | | | |
| ***GI symptoms*** | 3.93 | 0.001 | 1.75 - 8.82 | ***GI symptoms only*** | 4.26 | 0.008 | 1.45 - 12.50 |
| ***Age*** | 1.07 | 0.000 | 1.04 - 1.10 | ***Age*** | 1.08 | 0.000 | 1.04 - 1.12 |
| ***Being female*** | 0.18 | 0.000 | 0.08 - 0.40 | ***Being female*** | 0.22 | 0.001 | 0.09 - 0.54 |
|  | | | |  | | | |
| ***2001-2008 (31 deaths)*** | | | | ***2001-2008 (28 deaths)*** | | | |
| ***GI symptoms*** | 1.66 | 0.197 | 0.77 - 3.57 | ***GI symptoms only*** | 3.93 | 0.003 | 1.61 - 9.59 |
| ***Age*** | 1.03 | 0.039 | 1.00 - 1.05 | ***Age*** | 1.03 | 0.038 | 1.00 - 1.05 |
| ***Being female*** | 0.84 | 0.627 | 0.41 - 1.71 | ***Being female*** | 0.80 | 0.561 | 0.37 - 1.72 |

**Supplement table 5**

*Proportion dead within 28 days for 416 adult patients with invasive pneumococcal disease at admission to Aker University Hospital between 1993-2008 by sex and time period.*

| Time period | Proportion (%) dead within 28 days | | p-value |
| --- | --- | --- | --- |
|  | male | female |  |
| 1993-2008 | 18.6 | 11.6 | **0.045** |
| 1993-1999 | 27.9 | 10.9 | **0.004** |
| 2000-2008 | 13.0 | 12.1 | 0.827 |
